# Effectiveness of the introduction of a Chronic Care Model-based program for type 2 diabetes in Belgium

**DOI:** 10.1186/1472-6963-10-207

**Published:** 2010-07-14

**Authors:** Patricia Sunaert, Hilde Bastiaens, Frank Nobels, Luc Feyen, Geert Verbeke, Etienne Vermeire, Jan De Maeseneer, Sara Willems, An De Sutter

**Affiliations:** 1 Department of General Practice and Primary Health Care, Ghent University, Ghent, Belgium; 2 Department of General Practice, Interdisciplinary Healthcare and Geriatrics, Antwerp University, Antwerp, Belgium; 3 Department of Endocrinology, OLV-Hospital, Aalst, Belgium; 4 Leuven Statistics Research Centre, Leuven University, Belgium

## Abstract

**Background:**

During a four-year action research project (2003-2007), a program targeting all type 2 diabetes patients was implemented in a well-defined geographical region in Belgium. The implementation of the program resulted in an increase of the overall Assessment of Chronic Illness Care (ACIC) score from 1.45 in 2003 to 5.5 in 2007. The aim of the follow-up study in 2008 was to assess the effect of the implementation of Chronic Care Model (CCM) elements on the quality of diabetes care in a country where the efforts to adapt primary care to a more chronic care oriented system are still at a starting point.

**Methods:**

A quasi-experimental study design involving a control region with comparable geographical and socio-economic characteristics and health care facilities was used to evaluate the effect of the intervention in the region. In collaboration with the InterMutualistic Agency (IMA) and the laboratories from both regions a research database was set up. Study cohorts in both regions were defined by using administrative data from the Sickness Funds and selected from the research database. A set of nine quality indicators was defined based on current scientific evidence. Data were analysed by an institution experienced in longitudinal data analysis.

**Results:**

In total 4,174 type 2 diabetes patients were selected from the research database; 2,425 patients (52.9% women) with a mean age of 67.5 from the intervention region and 1,749 patients (55.7% women) with a mean age of 67.4 from the control region. At the end of the intervention period, improvements were observed in five of the nine defined quality indicators in the intervention region, three of which (HbA1c assessment, statin therapy, cholesterol target) improved significantly more than in the control region. Mean HbA1c improved significantly in the intervention region (7.55 to 7.06%), but this evolution did not differ significantly (p = 0.4207) from the one in the control region (7.44 to 6.90%). The improvement in lipid control was significantly higher (p = 0.0021) in the intervention region (total cholesterol 199.07 to 173 mg/dl) than in the control region (199.44 to 180.60 mg/dl). The systematic assessment of long-term diabetes complications remained insufficient. In 2006 only 26% of the patients had their urine tested for micro-albuminuria and only 36% had consulted an ophthalmologist.

**Conclusion:**

Although the overall ACIC score increased from 1.45 to 5.5, the improvement in the quality of diabetes care was moderate. Further improvements are needed in the CCM components delivery system design and clinical information systems. The regional networks, as they are financed now by the National Institute for Health and Disability Insurance (NIHDI), are an opportunity to explore how this can be achieved in consultation with the GPs. But it is clear that, simultaneously, action is needed on the health system level to realize the installation of an accurate quality monitoring system and the necessary preconditions for chronic care delivery in primary care (patient registration, staff support, IT support).

**Trial Registration:**

Trial registration number: ClinicalTrials.gov Identifier: NCT00824499

## Background

During the last decade many countries have taken the first steps towards the adaptation of their health care system to a more chronic care oriented system and often the Chronic Care Model (CCM) has been used as a conceptual framework to design the interventions [1]. Most knowledge on the effect of the introduction of CCM elements on the quality of chronic care originates from organizations or countries with a well-structured primary health care system. Information about efforts made in other health care systems is still scarce [2,3]. This study reports on the effectiveness of a CCM-based intervention in a high-income country with good health care facilities but with a primary health care system with limited structure.

In 2003, the Belgian National Institute for Health and Disability Insurance (NIHDI) commissioned a pilot study to explore how chronic care could be organized in a more efficient way in the Belgian health care setting. In the Belgian context, efforts to support patients with chronic diseases are mainly hospital-centered and most chronic disease patients in primary care are still seen in the context of a system focused on acute care [4,5]. In order to explore ways to adapt the primary health care system to a more chronic care oriented system, we set up a four-year action research project (1^st ^July 2003 - 30^th ^June 2007) in a well-defined geographical area [6]. Although the study focused primarily on type 2 diabetes, it was our intention to develop an approach, applicable to other chronic diseases in primary care. The main characteristics of the Belgian health care system are summarized in table 1[7,8].

**Table 1:** Main characteristics of the Belgian health care system (2004)

• Financing basis of public health is contribution and tax based
• Health expenditure amounts to 9.3% of gross domestic product (GDP)
• Health care expenditure per capita is 2922 (in US$ PPP)
• Coverage of population by public health is almost universal (compulsory)
• Out of pocket payments (proportion of total health expenditure) equals 23%
• Payment of GP is mainly fee-for-service with a limited capitation fee for registered patients
• GP to population ratio 1:909
• GP has no gatekeeper function; voluntary patient registration with a GP (since 1999)
• Approximately 70% of the GPs are working in single-handed practices
• About 66% of the GPs are using an electronic medical record (EMR)

Equity and freedom of choice are high priorities for health policy. There is no clearly defined gatekeeper function in place; every citizen has free access to medical specialists and hospital care, even as the first point of contact with the health system. Most care providers work as independent self-employed health professionals. The patient pays directly to the care provider and is entitled to a reimbursement by his sickness fund. Most services are reimbursed at a rate of 75%. In general, patient satisfaction with the health care system is high [9]. Diabetes teams in primary care are mostly loose networks of single-handed care providers. Patients on insulin therapy have the possibility to register in the hospital where they receive care from a multidisciplinary team (diabetologist, diabetes educator, dietician). In 2003, the region counted 83 GPs (GP to population ratio of 1:972), 70% of whom worked in a single-handed practice, most of them without any support staff. Structured self-management support programs were not in place in primary care. Further, there was little or no coordination regarding diabetes care delivery in the region and there were no formal shared care models in place.

We decided to define priorities for change in consultation with the local care providers (primary and secondary care) in order to enhance the implementation and the sustainability of the intervention. In 2003 a written survey, assessing the strengths and the weaknesses of the organization of diabetes care was conducted among all care providers in the region (GPs, specialists, pharmacists, dieticians, podiatrists, nurses, home care) involved in diabetes care. The survey results were discussed with representatives from the different disciplines (study group meetings). Two main issues arose from the discussions: the need for more coordination and continuity of care and the need for more patient support. In consultation with the region a complex intervention was subsequently developed and introduced progressively in the region (table 2).

**Table 2:** Introduction of the CCM components in the region (1^st ^July 2003 - 30^th ^June 2007)

**• Organization of the Healthcare Delivery System**
-July 2003: installation of a local steering group
-July 2004: appointment of a program manager
**• Community Linkages**
-Continuously 2003-2007
**• Self-Management Support**
-October 2004: individual education program
-June 2005: group-based education program [10]
**• Decision Support**
-September 2004: distribution of type 2 diabetes guideline
-Continuously 2004-2007: provider education
**• Delivery System Design**
-December 2004- June 2005: development of an interdisciplinary care protocol
-February 2005: support program for the initiation of insulin therapy
**• Clinical Information Systems**
-September 2004 - January 2005: regional audit -June 2005: regional feedback

The Chronic Care Model was used as a conceptual framework for priority setting, development and evaluation of the program. The successive steps of the model for Accelerating Improvement, a scientific method used for action-oriented learning, were followed and an implementation strategy was developed based on the current evidence on the successful implementation of care innovation [11].

**Figure 1:**
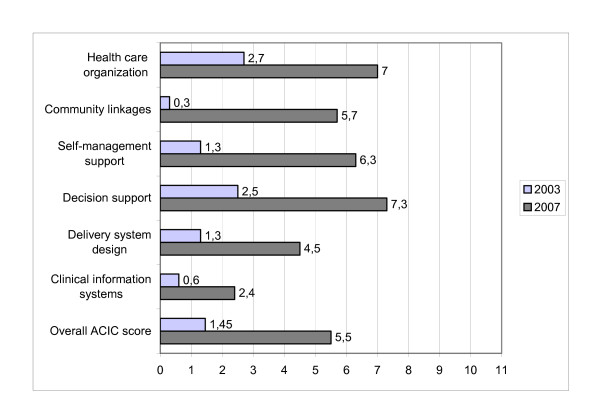
Assessment of Chronic Illness Care (ACIC) in the intervention region* (2003-2007). *The highest score ("11") indicates optimal support for chronic illness, the lowest score "0" corresponds to limited support.

The implementation of the CCM components in the intervention region was assessed in 2007 by the Assessment of Chronic Illness Care (ACIC) survey [12]. The ACIC survey provides scores corresponding to each of the six CCM components. In the ACIC, the highest score ("11") indicates optimal support for chronic illness, the lowest score ("0") corresponds to limited support for chronic illness. The efforts made in the region resulted in an increase of 4.05 points in the overall ACIC score, indicating a shift towards a more chronic care-centered system. Figure 1 gives an overview of the scores achieved on the different CCM components.

In accordance with the needs expressed by the region, clear progress has been made in aspects related to organization of health care and self-management support. The scores for community linkages and decision support progressed in a similar way. Although these components were not the main focus of the project the scores increased as roles in diabetes care were redefined. Consequently, linkages with socio-cultural organizations (e.g. diabetes patients' organization) were strengthened and the specialists' role has evolved towards that of a primary care coach. The scores for delivery system design and clinical information systems improved the least.

In 2007 we were not able to report on the relation between the introduction of CCM components and the quality of diabetes care achieved in the region. The data on quality of care, collected in a real-life setting, were useful to guide the process of priority setting but not valid enough to evaluate the effect of the intervention in the region. In order to evaluate the effectiveness of the intervention, a follow-up study was financed by the NIHDI. We reported earlier on the opportunities and barriers experienced during the implementation of the intervention in the region [13]. The current paper reports on the effect evaluation.

The paper addresses the following questions:

1. Does the implementation of a CCM-based program for type 2 diabetes improve the delivery of care (process) in the intervention region compared to the control region where usual care continues?

2. Does the implementation of a CCM-based program for type 2 diabetes improve the quality of care (intermediate outcome) in the intervention region compared to the control region where usual care continues?

## Methods

### Study design

A quasi-experimental design involving a comparable control region was chosen to evaluate the effect of the intervention in the region [14]. The control region was selected based on similar geographical and socio-economic characteristics and comparable health care facilities as the intervention region. Table 3 summarizes the main characteristics of the intervention and control region in 2003 [15].

**Table 3:** Baseline characteristics of the intervention and the control region (2003)

	Intervention region	Control region
**Geographical**		
Inhabitants	76,799	68,663
Population density (n/km²)	984	827
**Socio-economic**		
Female population (%)	50.8	50.6
Mean age (years)	41,32	40.63
Population over 65 years (%)	18.1	16.2
Foreigners (%)	1.9	3.5
Employment rate (men) (%)*	75.2	74.9
Employment rate (women) (%)*	61.5	58.5
Mean income (%)**	104.7	98.4
**Health care facilities**		
GP to population ratio	1:972	1:1069
Hospitals in the region	2	2
Number of endocrinologists	3	2

*Proportion of men and women employed in relation to the available workforce in the region.

**Mean income in relation to the mean income in Flanders.

### Data sources

In collaboration with the InterMutualistic Agency (IMA), an organization in which all Belgian Sickness Funds (n = 7) are represented, and the laboratories from both regions, a research database was set up in 2008. Data collection and data cleaning were executed following standardized procedures. The data management process was coordinated by a senior data manager appointed to the University of Antwerp (PB) and two members of the research team (HB, CVDB).

In order to obtain information about population characteristics, medication use, health care consumption (process of care) and health care costs, we cooperated with the IMA. In Belgium, all individuals entitled to health insurance (almost 99% of the population) have to register with a sickness fund. The services covered by the compulsory health insurance are described in a nationally established fee schedule and reimbursement of health care costs is linked to specific codes for each service delivered. An HbA1c test, for example, is registered as code 540750. By extracting the codes for a number of predefined services we were able to assess the process indicators. In our study, Sickness Fund data were selected for each patient born in 1966 or earlier, living (at least for some time) in the region between 1^st ^January 2004 and 31^st ^December 2006 and meeting at least one of the following criteria for the period 2002-2006: reimbursement of at least one HbA1c test, reimbursement of diabetes-specific medication, registration in a hospital-based diabetes centre. VDV coordinated the IMA data collection.

In order to obtain information about intermediate outcomes (HbA1c, total cholesterol) we invited the laboratories from both regions (ambulatory and hospital-based) to provide data. Ten (six in the intervention and four in the control region) of the eleven invited laboratories, agreed to participate. The non-participating laboratory owned only data from a small number of patients referred by one GP (control region). The laboratories were asked to provide data from each patient living (at least for some time) in the region between 1^st ^January 2004 and 31^st ^December 2006 and having at least one HbA1c measurement performed during the period 2002-2006. CVDB coordinated the data collection process among the laboratories.

The laboratory data were subsequently linked to the IMA data by a Trusted Third Party (TTP) using a unique identification code. The process of data extraction, data linking and data analysis was approved by the Sectoral Committee of Social Security and Health and by the Commission for the protection of the Privacy (authorization 08/05/07, 06/05/2008 and 18/08/2008; SCSZ/07/076) [16].

### Study population

The intervention population included all type 2 diabetes patients in the defined area. Since the Sickness Funds do not register diagnostic information, we had to identify persons with type 2 diabetes using indirect criteria for diabetes (table 4) [17]. To make sure that persons selected in the cohort were "real" type 2 diabetes patients, we only included patients who were on antidiabetic medication in 2003. Patients treated with 3 or more insulin injections a day in 2003 were excluded from the study cohort in order to exclude most of the diabetes type 1 patients given the fact that they were not the target group of this intervention. In 2003, on average, 84% of the diabetes type 1 patients in Belgium were treated with 3 or more insulin injections (personal communication ND, Institute of Public health (IPH)) [18].

**Table 4:** Criteria used to extract the study cohort

Inclusion criteria
-living in the region in 2004
-born in 1966 or before (≥40 years in 2006)
-reimbursement of at least one prescription for antidiabetic agents (oral therapy, insulin therapy) in 2003 (year before the start of the intervention)
**Exclusion criteria**
-≥3 daily insulin injections (before 01/01/2004)

Diabetes patients who moved to a more complex insulin scheme during the study period were not excluded. To make the exposure time to the intervention comparable for all patients, we only included persons who were exposed to the intervention from the start (patients living in the region in 2004). Persons who moved or died during the intervention period were not excluded from the study cohort. Patients who started antidiabetic therapy after 1^st ^January 2004 were excluded in order to limit bias by the inflow of patients whose treatment had been adjusted in consequence of the intervention.

### Principal outcomes

We defined a set of quality indicators, six process and three outcome indicators, to evaluate the effect of the intervention in the region. To define the set of quality indicators we relied on the indicators currently used in the Quality and Outcomes Framework (QOF) and the availability of the data in the research database (table 5) [19,20].

**Table 5:** Set of nine quality indicators

Quality indicator	Data source
**Indicators of care process**	IMA
▪ The percentage of patients with at least one assessment of HbA1c during the last 12 months	
▪ The percentage of patients with at least one assessment of cholesterol during the last 12 months	
▪ The percentage of patients with a prescription for statin therapy during the last 12 months	
▪ The percentage of patients with at least one assessment of micro-albuminuria during the last 12 months	
▪ The percentage of patients with a prescription for influenza vaccination during the last 12 months	
▪ The percentage of patients with at least one assessment by an ophtalmologist during the last 12 months	
**Indicators of intermediate outcome of care**	Laboratories
▪ The percentage of patients with diabetes in whom HbA1c is ≤7.5%	
▪ The percentage of patients with diabetes in whom HbA1c is ≤10%	
▪ The percentage of patients with diabetes whose total cholesterol is ≤190 mg/dl	

We included the prescription of statin therapy as a process indicator given the fact that cardiovascular risk prevention was a priority for change in the region. The prescription of an influenza vaccination (also included in the QOF) was added in order to evaluate the attention for the overall prevention strategies in the region. The cutoffs of the outcome quality indicators were defined according to the cutoffs currently used in the Quality and Outcomes Framework (QOF).

The process indicators were defined using the reimbursement codes in the Sickness Fund data. Concerning the intermediate outcome indicators, the data provided by the laboratories were used. All HbA1c and total cholesterol tests performed during the intervention period (real-life measurements) were included in the analysis. Since 2002, Belgian laboratories participate in an External Quality Assessment (EQA) program, organized by the Scientific Institute of Public Health (IPH) [21]. All participating laboratories use Diabetes Control and Complications Trial (DCCT)-aligned methods to determine HbA1c and since 2004, the performance quality of Hba1c testing has been good to very good in all laboratories involved. The determination of total cholesterol is less prone to measurement errors, and for this reason total cholesterol test results can be used for analysis purposes since 2002 (personal communication CVC, IPH).

### Statistical analysis

Statistical analysis was supervised and performed by members (AC, AI, GV) of the Leuven Statistics Research Centre, an institution experienced in longitudinal data analysis.

Baseline characteristics of the diabetes population in both regions were compared using a t-test for continuous variables and a Chi-squared test for dichotomous variables respectively. Means and standard deviations were reported for continuous variables, proportions for dichotomous variables.

In order to study the evolution of the dichotomous process indicators in both regions, a Generalized Estimating Equations (GEE) model was used [22]. This model accounts for the clustering of data within patients (repeated measures). In the model, the achievement of a specific target in one year (e.g. HbA1c test performed in 2006), was the dependent variable, whereas time (before and after the intervention period) and group (intervention *vs*. control region) were the independent variables. The evolution in both regions was compared using a test for interaction between time and region.

Continuous outcome indicators (HbA1c and total cholesterol) were analyzed with linear mixed models, with the patient as random effect [23]. All patients, for whom at least one HbA1c and/or total cholesterol measurement were available during the study period, were included in the analysis. The analysis of HbA1c measures included 80% of the patients, the analysis for cholesterol measures 77% of the patients. For those patients, all HbA1c and total cholesterol measurements received from the laboratories during the study period ("real life" measurements) were included. In the model, logarithmic transformed HbA1c measurements and total cholesterol measurements were the dependent variables, time and group (intervention vs. control region) the independent variables. Comparison of the evolution in both regions was done using a test for interaction between time and region.

All analyses were performed using SAS (version 9.2) and a significance level of 5% was used. Evaluations were done according to the intention-to-treat principle.

## Results

### Baseline characteristics of the diabetes population in the intervention and control region

In total 4,174 persons who met the defined criteria were selected from the database; 2,425 patients (52.9% women) with a mean age of 67.5 from the intervention region and 1,749 patients (55.7% women) with a mean age of 67.4 from the control region. Table 6 summarizes the baseline characteristics of both study cohorts.

**Table 6:** Baseline characteristics of the type 2 diabetes populations (2003)

	Intervention region N = 2,425	Control region N = 1,749	P value*
% female population	52.87	55.69	0,071
Mean age (years)	67.54 ± 11.08	67.40 ± 10.73	0,692
% ≥75	28.46	27.73	0,608
% reduced co-payments	32.91	31.62	0,380
% registered with GP	62.39	65.01	0,083
% registered in diabetes centre	5.3	3.9	0.039
% insulin therapy	15.67	15.44	0,838
Mean HbA1c (01/01/2004)	7.55(7.48-7.62)	7.44(7.34-7.53)	0.057
Mean total cholesterol (01/01/2004)	199.07(197.01-201.13)	199.44(197.03-201.90)	0.812
% hospitalisation during the last year	23.71	24.71	0,600

*t-test for continuous variables; Chi-square test (MH Chi-Square) for dichotomous variables

There were no statistically significant differences regarding, demographic, socio-economic and diabetic characteristics at the baseline. Both study groups only differed significantly regarding the percentage of patients registered in a hospital-based diabetes centre, although the absolute difference was small.

### Evolution of the process indicators

Table 7 summarizes the evolution of the process indicators from baseline until the end of 2006.

**Table 7:** Changes in process indicators°

	% of patients receiving services		Evolution 2003-2006	
	**2003**	**2006**	**P value for the Δ** in both regions *	**P value for the Δ** between both regions**

**HbA1c test**				
Intervention region	65.52	71.25	+5.73 (p < 0.0001)	p = 0.0117 (1)
Control region	68.58	70.60	+2.02 (p = 0.0580)	
**Total Cholesterol test**				
Intervention region	72.53	73.33	+0.80 (p = 0.3888)	p = 0.0190 (1)
Control region	74.21	69.14	-2.59 (p = 0.0178)	
**Statin therapy**				
Intervention region	27.07	45.15	+18.08 (p < 0.0001)	p = 0.0002 (1)
Control region	25.15	37.54	+12.39 (p < 0.0001)	
**Micro-albuminuria test**				
Intervention region	20.57	25.96	+5.39 (p < 0.0001)	p = 0.1325
Control region	15.71	18.34	+2.63 (p = 0.0037)	
**Influenza vaccination**				
Intervention region	57.43	58.14	+0.71 (p = 0.4627)	p = 0.0462 (2)
Control region	50.35	53.91	+3.56 (p = 0.0007)	
**Ophthalmologist visit**				
Intervention region	34.72	36.10	+1.38 (p = 0.1360)	p = 0.9300
Control region	31.18	32.36	+1.18 (p = 0.2653)	

°GEE model; Δ = evolution; *P value for the evolution (2003-2006) in the intervention and control region; **P value for the evolution (2003-2006) between the intervention and control region, (1) significantly greater change in the intervention region (2) significantly greater change in the control region

The proportion of patients with at least one HbA1c assessment in the last 12 months increased significantly in the intervention region, although the improvement was moderate. Significantly more patients had their urine tested for micro-albuminuria in 2006, but this evolution was not significantly different from the one in the control region. Most progress was made in the proportion of patients who were prescribed statin therapy. Cardiovascular risk assessment and the prescription of statin therapy according to the guidelines were priorities for change in the region. Both in the intervention and in the control region, significantly more patients were prescribed statin therapy in 2006, although the rise was significantly greater in the intervention region (p = 0.0002). The other process indicators remained almost status quo. In 2006, as in 2003, significantly more patients in the intervention region (p = 0.0061) received an influenza vaccination. However, the control region evolved better (p = 0.0462). According to the IMA data 94% of the patients received at least one HbA1c test during the study period, 96% at least one cholesterol test. These figures reflect that there are little or no patients who receive no care at all. However, the percentage of patients receiving an annual review as recommended in the guidelines remains low. Especially the assessment of long-term diabetes complications remains suboptimal.

### Evolution of the intermediate outcome indicators

Mean HbA1c and cholesterol levels decreased significantly during the study period, both in the intervention and in the control region (p < 0.001; table 8). The decrease in mean cholesterol level was significantly greater in the intervention region (p = 0.0021).

**Table 8:** Changes in intermediate patient outcomes°

	Intervention period		Δ 01/01/04-01/01/07	P value*
	**01/01/2004**	**01/01/2007**		

**HbA1c (%)**				
Intervention	7.55(7.48-7.62)	7.06(7.00-7.12)	-0.49(p < 0.0001)	0.4207
Control	7.44(7.34-7.53)	6.90(6.83-6.98)	-0.54(p < 0.0001)	
**TC (mg/dl)**				
Intervention	199.07(197.01-201.13)	173.94(171.85-176.04)	-25.13(p < 0.0001)	0.0021 (1)
Control	199.44(197.03-201.90)	180.60(177.83-183.41)	-18.84(p < 0.0001)	

°linear mixed model; Δ = evolution; *P value for the evolution between the intervention and control region, (1) significantly greater change in the intervention region (2) significantly greater change in the control region

The improvement in mean HbA1c and cholesterol levels was reflected in the percentage of patients achieving intermediate outcome targets at the end of 2006 (table 9).

**Table 9:** Changes in intermediate outcome indicators°

	% of patients achieving targets		Evolution	
	**01/01/04**	**01/01/07**	**P value Δ** in both regions *	**P value Δ** between both regions**

**HbA1c ≤ 7,5%**				
Intervention	55.40	61.68	+6.28 (p < 0.0001)	0.3425
Control	56.67	65.04	+8.37 (p < 0.0001)	
**HbA1c ≤ 10%**				
Intervention	93.13	93.89	+0.76 (p = 0.3561)	0.1479
Control	93.63	95.94	+2.31 (p = 0.0184)	
**TC ≤ 190 mg/dl**				
Intervention	40.49	58.81	+18.32 (p < 0.0001)	0.0305 (1)
Control	41.15	53.95	+12.80 (p < 0.0001)	

°GEE model; Δ = evolution; *P value for the evolution in the intervention and control region; **P value for the evolution between the intervention and control region, (1) significantly greater change in the intervention region (2) significantly greater change in the control region

One of the priorities in the intervention region was to increase the proportion of patients with an HbA1c value of 7.5 or less. In 2006, there was a significant increase in patients achieving an HbA1c level of 7.5 or less in the intervention region (p < 0.0001) but the progression was not significantly different from the one in the control region. The proportion of patients achieving a cholesterol level of 190 mg/dl or less increased significantly more in the intervention region. Most probably, this evolution was due to the significant increase in statin use.

## Discussion

### Summary of main findings

This study reports on the effect of an action research project (complex intervention; table 2) on the quality of care for type 2 diabetes patients. At the end of 2006 improvements were observed in five of the nine defined quality indicators in the intervention region, three of which (HbA1c assessment, statin therapy, cholesterol target) improved significantly more than in the control region. The systematically assessment of long-term diabetes complications (nephropathy and retinopathy screening) remained insufficient. In 2006 only 26% of the patients had their urine tested for micro-albuminuria and only 36% had consulted an ophthalmologist. Although the progression was moderate, there may be room for optimism, given the fact that the effect of the intervention was not yet at its maximum. Due to the chosen methodology, i.e. action research, the different components of the intervention were introduced progressively in the region and participation of patients and care providers increased gradually.

### Strengths of the study

Quality data are scarce in Belgium and regular monitoring of diabetes care is limited to patients registered in a hospital-based diabetes centre [24,25]. A key strength of the study is that, for the first time in Belgium, quality data were available for all type 2 diabetes patients in a well-defined region. As a consequence of the use of the IMA data to define the study cohorts, recruitment bias was prevented. In this way the data reflect "real-life" quality of diabetes care achieved for diabetes patients on medication.

Another important strength of the study is the availability of data from a control region with similar geographical and socio-economic characteristics and health care facilities comparable to those in the intervention study. The chosen study design allows for a comparison of the evolution in the intervention region with the evolution in a control region, where usual care was continued. In this way time trends could be taken into account.

### Limitations of the study

The Sickness Funds do not register diagnostic information. For this reason diabetes patients could only be selected through indirect criteria. As a consequence the study gives no information about type 2 diabetes patients treated with lifestyle therapy. Furthermore, we excluded patients treated with 3 or more insulin injections a day (8.6% of the diabetes patients on medication in 2003), in order to limit the presence of type 1 diabetes patients in the study cohorts. In doing so we inevitably also excluded type 2 diabetes patients treated with an intensive insulin scheme, accounting for about a quarter of the type 2 patients treated with insulin in 2003 (personal communication ND, IPH). Diabetes patients who moved to a more complex insulin scheme during the study period were not excluded. This information is of particular interest when our study results are compared with these of other study populations.

However, we assume that the limitations related to the inclusion and exclusion of patients were comparable for both study cohorts and have not affected the main conclusions. Moreover, the prevalence of type 2 diabetes in both study cohorts is very similar to the prevalence recorded in previous studies in Belgium, indicating that no systematic fall out of patients occurred [26].

The use of administrative data to define the quality indicators, implies that the current study gives no information about some important quality indicators only available via medical records (e.g. smoking, weight control and blood pressure). The data, used to define the quality indicators, are administrative data, primarily meant to reimburse patients and care providers. During the process of recoding these data, some errors may have occurred. Again, there is no reason to presume that this is different for the two study cohorts. Another limitation of the use of administrative data is the delay with which these data are available for evaluation. In Belgium, patients and care providers can declare their health care bills until two years after the service has been delivered. For this reason the study could not start until 2008.

### Results in relation to the CCM elements targeted

The ultimate purpose of the study was to inform health policy leaders about effective strategies to adapt primary health care to a more chronic care oriented system. In this light the moderate gain in quality of diabetes care in relation to the 4.05 progress in the overall ACIC score needs some reflection. Although the evaluation of a complex intervention is challenging given the fact that components of the intervention may act both independently and interdependently, we tried to explain the study results in relation to the different CCM components targeted in the intervention [27]. More insight in this relationship is also crucial in the light of future actions. There is a growing body of literature on the relation between investments in components of the CCM and effects on quality of chronic care [28-30]. The rather limited progress in quality of care in relation to the 4.05 progress in the overall ACIC score can probably be explained on the one hand by the nature of the elements of the CCM we have targeted (Figure 1) and by the chosen methodology (action research) on the other hand. In accordance with the needs expressed by the region we have spent a lot of effort strengthening primary care at the meso level (program manager, steering group, study groups), which resulted in a clear increase of the ACIC scores for organization of health care and community linkages. These efforts were indispensable for the coordination of diabetes care in the region and the facilitation of the quality improvement process but probably did not have a direct effect on the quality of care at the patient level. The investments in decision support (guideline dissimination, provider education, regional feedback, support by specialists) have certainly raised the awareness regarding the need for a more stringent therapeutic approach of diabetes (timely upgrading of diabetic medication and cardiovascular risk assessment). Mean HbA1c and lipid levels decreased significantly in the intervention region during the study period. The similar evolution of the HbA1c levels in the control region can probably be explained by a time trend. The guideline type 2 diabetes, first introduced in the intervention region, was introduced in all regions in Flanders in 2005, accompanied by an intensive information campaign. Regarding the evolution of the lipid levels, there is an indication that the positive trend was already ongoing before the study started (unpublished data). The intervention has given an extra boost regarding statin prescription. In consultation with the region an education program, integrated in primary care, was launched for diabetes patients on lifestyle and/or medication therapy. The potential benefit of the introduction of the program in the region is probably underestimated in the selected study cohort. On the one hand, due to the kind of data available for evaluation, an important target group for the education program, i.e. newly diagnosed patients, was excluded from the current study cohort. On the other hand, due to the methodology of action research, the participation of patients increased gradually and the effect of the program was certainly not yet at its maximum. The project had only a limited impact on the organization of diabetes care at the practice level (delivery system design) and the use of information technology (IT) (clinical information systems) in practice, e.g. in 2007 30% of the GPs mentioned to have some diabetes register (mostly on paper), but this list was not routinely used to plan follow-up or other actions (survey among GPs, 2007). The limited progress in these components probably explains the poor results regarding the assessment of long-term diabetes complications.

### Quality of care in relation to international trends

As mentioned above, this is the first time that quality data were available for all type 2 diabetes patients from two regions in Belgium. As a result these data reflect the quality of care achieved in a country where initiatives to support chronic care were, until recently, mainly hospital-based and where primary care teams are usually flexible and loose networks of single-handed care providers [31].

A comparison of the baseline data of a recent review of published observational studies in primary care (same time span as our study) with the baseline data of our study, revealed similar results regarding intermediate outcome indicators [32]. As to the process indicators, the same trends were observed as in our study. The standard of care achieved for the more complex process indicators (e.g. retinal screening) was lower than for the simple process indicators (e.g. HbA1c assessment), although in general our scores were lower. This can partly be explained by the fact that in our study compliance was assessed over the previous 12 months while this was 15 months in most of the studies cited in the review.

Another interesting comparison source is a study evaluating the effect of the QOF on diabetes care in a whole county (Shropshire) in the same time span as our study [33]. In 2004, similar quality data were observed as in our study. In 2006, two years after the introduction of the QOF, the quality data improved substantially more than in our study (e.g. HbA1c recording increased from 75% to 94%; retinal screening from 47% to 84%). The QOF, a pay-for-performance scheme based on meeting targets for the quality of clinical care, was introduced in England in 2004. In general this scheme has accelerated quality improvement in different aspects of clinical care, although some authors also warn against unintended consequences, including reductions in the quality of care not linked to incentives and in the continuity of care [34]. The introduction of the QOF was accompanied by important investments in primary care [35]. Furthermore, in England, primary care has a long tradition of multidisciplinary teams and patients are registered with a specific primary care facility, factors which facilitate quality improvement.

### Implications for health policy and future research

The results of the current study reflect the fact that the project succeeded in strengthening primary care at the meso level but had only limited impact on the organization of care at the practice level (delivery system design and clinical information systems). This became especially apparent in the inadequate monitoring of long term complications. The quality level observed in the intermediate outcomes is acceptable, which reflects the continuous investments in quality improvement at provider level in Belgium [36]. However, there is still room for improvement (e.g. +/- 25% of the patients has an HbA1c level ≥8% in 2006). Comparable results have been seen in other settings starting to adapt their care system to a more chronic care oriented system as e.g. described in an evaluation of CCM implementation in primary care in the US (Minneapolis-St Paul Metropolitan region). Implementing the CCM elements in a system originally set up to deliver acute and episodic care, takes time. It demands to switch the focus of quality improvement efforts from the individual level of patients and care providers to the system level. Further, strong leadership and a careful change management are continuous needed [37]. Future research initiatives in the Belgian context need to explore how the practice level can be adapted to a more chronic care oriented approach. The regional network, as established in our project, is an ideal platform to start up further quality improvement projects. The NIHDI has recently decided to finance the establishment of comparable networks in other regions in Belgium. Currently (since July 2009) several regions have started to appoint program managers.

We already reported on the opportunities and barriers regarding chronic care organization in the Belgian context, and emphasized the need for clear action on the health system level regarding the installation of a quality monitoring system on the one hand and the strengthening of recent initiatives to facilitate chronic care delivery in primary care (patient registration, staff support, IT support) on the other hand. These are essential preconditions for adequate chronic care.

The effect of the introduction of an education program for diabetes patients in the region is probably not yet measurable in this study cohort. One of the main researchers (HB) is currently evaluating the effect of the education program on the quality of care among patients who attended the program. Future research needs to explore how this program can best be continued in primary care.

## Conclusion

This study reports on the relation between the implementation of CCM elements and the quality of diabetes care achieved in a country where the efforts to adapt primary care to a more chronic care oriented system are still at a starting point. Although the overall ACIC score increased from 1.45 to 5.5, the improvement in the quality of diabetes care was moderate. Strengthening primary care at the meso level contributed to a better coordination of diabetes care, but probably did not influence the quality of care at the patient level. The project had only a limited impact on the organization of diabetes care at the practice level (delivery system design) and the use of information technology (IT) (clinical information systems) in practice. Future research initiatives in the Belgian context need to explore how the practice level can be adapted to a more chronic care oriented approach. The regional networks, as they are financed now by the NIHDI, are an opportunity to explore how this can be achieved in consultation with the GPs. But it is clear that, simultaneously, action is needed on the health system level to realize the installation of an accurate quality monitoring system and the necessary preconditions for chronic care delivery in primary care (patient registration, staff support, IT support).

## Competing interests

The authors declare that they have no competing interests.

## Authors' contributions

PS, HB, EV, JDM and GV participated in the development of the study design. PS and HB drafted the manuscript. PS, HB, FN, LF, GV, EV, JDM, SW and ADS supervised the data collection and analyses. All contributing authors have read and approved the final manuscript.

## Funding source

The project was funded by a research grant from the Belgian National Institute for Health and Disability Insurance (NIHDI) in Belgium.

## Pre-publication history

The pre-publication history for this paper can be accessed here:

http://www.biomedcentral.com/1472-6963/10/207/prepub
